# On the origins of strain inhomogeneity in amorphous materials

**DOI:** 10.1038/s41598-018-19900-2

**Published:** 2018-01-25

**Authors:** Alexander J. G. Lunt, Philip Chater, Alexander M. Korsunsky

**Affiliations:** 1 CERN (European Council for Nuclear Research), CH-1211 Geneva 23, Switzerland; 2 Diamond Light Source, Harwell Science and Innovation Campus, Didcot, OX11 0QX United Kingdom; 3 Multi-Beam Laboratory for Engineering Microscopy, Department of Engineering Science, University of Oxford, Parks Road, Oxford, OX1 3PJ United Kingdom

## Abstract

Strain is a crucial measure of materials deformation for evaluating and predicting the mechanical response, strength, and fracture. The spatial resolution attainable by the modern real and reciprocal space techniques continues to improve, alongside the ability to carry out atomistic simulations. This is offering new insights into the very concept of strain. In crystalline materials, the presence of well-defined, stable atomic planes allows defining strain as the relative change in the interplanar spacing. However, the presence of disorder, e.g. locally around defects such as dislocation cores, and particularly the pervasive atomic disorder in amorphous materials challenge existing paradigms: disorder prevents a reference configuration being defined, and allows strain to be accommodated in a different manner to crystalline materials. As an illustration, using experimental pair distribution function analysis in combination with Molecular Dynamic (MD) simulations, we highlight the importance of bond angle change vs bond stretching for strain accommodation in amorphous systems.

## Introduction

Amorphous materials differ from their crystalline counterparts by the absence of long-range order. This means that even when the local environment of the atom of a chosen type is consistent (short-range order), the same arrangement does not persist over a long enough distance to allow confident prediction of the location of another atom. At intermediate distances (medium-range order) randomisation takes place, whereby short-range order is lost, and long-range disorder is established.

A vast list of chemical, physical and mechanical properties of materials depend on their deformability, i.e. ways in which external influences force the atoms to change their relative positions (often in subtle ways, against the backdrop of thermal fluctuations). For example, the rotation, stretching or shortening of interatomic bonds have important implications for electronic interactions, functional properties and phase structure^1^.

X-ray crystallography is an indispensable tool for structural studies, allowing the determination of atomic positions with Ångstrom precision. The method relies on the strong constructive interference of scattered waves from a large number of nearly identical atomic planes placed at nearly identical interplanar distances, even when external load is applied. This is due to the fact that a long-range ordered structure has constant density of atomic packing and bonds, and may only accommodate strain in an affine (preserving plane parallelism) way, i.e. so that the displacement of each atomic plane is proportional to its distance from the chosen reference, and deformation is uniform over the same long-range domain considered. Furthermore, the Bragg scattering from these planes is not affected by the relative shear of atomic planes parallel to each other, associated with small changes in the bond angle.

However, the presence of disorder characteristic of amorphous materials allows a different mode of deformation to be adopted. An amorphous structure contains regions of lower and higher density of matter and interatomic bonds, meaning that some regions may deform more readily than others. Atoms may show a tendency to shift into low density regions away from high density locations. If one considers the stiffness of the atomic arrangement, then *local* bond rotation may occur more readily than bond stretching, meaning that it may play a crucial role in accommodating this inhomogeneous deformation mode. Further investigations are necessary in order to understand the length scales over which these two differing effects influence the macroscale strain response of a given material.

In terms of modelling approaches, Molecular Dynamics (MD) has a great potential to develop enhanced understanding of the deformation response of these systems^2,3^, however, considering the great degree of dependence of this approach upon the use of correct interatomic potentials and simulation conditions, they require experimental validation.

Few experimental techniques are capable of resolving strain accommodation methods in amorphous materials. Improved insights have been offered by spectroscopy based methods, such as Extended X-ray Absorption Fine Structure (EXAFS)^4^ and EXtended Electron Energy Loss Fine Structure (EXELFS)^5^. These methods are capable of extracting pair distribution functions between atoms, but are not able to resolve the directional (azimuthal) variation. Atomic Pair Distribution Function (PDF) analysis based on X-ray scattering data provides information about interatomic distances in non-crystalline materials that is sufficiently precise to allow the determination of elastic strain^6^, and, as shown below, is able to resolve its dependence on direction. In the present study we consider the effects of stress and strain on the atomic pair-wise radial distribution function in amorphous silica. Both tensile and compressive loading regimes were examined up to a maximum stress of approximately 300 MPa in order to investigate the typical residual and dynamic loads present within a ceramic structural component. We found that in the deformed material, near neighbour atoms invariably experience smaller strain (measured by the change in the distance between atoms) compared to the macroscopic value, whilst with increasing interatomic distances this strain approaches the macroscopic value. By considering molecular dynamics simulation of amorphous silica deformation, we demonstrate that the reason for the observed behaviour is the presence of relatively large (many degrees) change in the inter-bond angles. This ‘scissoring’ deformation mechanism offers a route for strain accommodation that is alternative to the elongation or shortening of the stiff bonds.

## Results and Discussion

High spatial resolution mapping of strain variation in highly disordered materials continues to present a major challenge, since well-established techniques such as micro-/nano-beam diffraction of X-rays and electrons fail to produce the sharp regular patterns that lend themselves readily to quantitative interpretation^7–9^. An alternative approach gaining recognition is the use of Focused Ion Beam to induce strain relief by material removal, and digital image correlation techniques that does not rely on the crystalline nature of the material^10,11^. Few examples are available in the literature of quantifying elastic strain in amorphous materials by diffraction^6,12,13^. There is a lack of clarity regarding the fundamental mode of deformation response of an amorphous atomic arrangement. In particular, although well documented, the crucial fact remains unexplained that nearest neighbour interatomic bonds appear much stiffer than the macroscopic material properties. Short range deformation within an amorphous structure must be accommodated by another mechanism that offers greater compliance. We surmise that bond rotation plays a crucial role in accommodating strain at the atomic scale, and demonstrate that this is indeed the case in amorphous silica as one of the most important amorphous substances that forms the basis of glasses, fibres, coatings, composites, etc.

**Figure 1:**
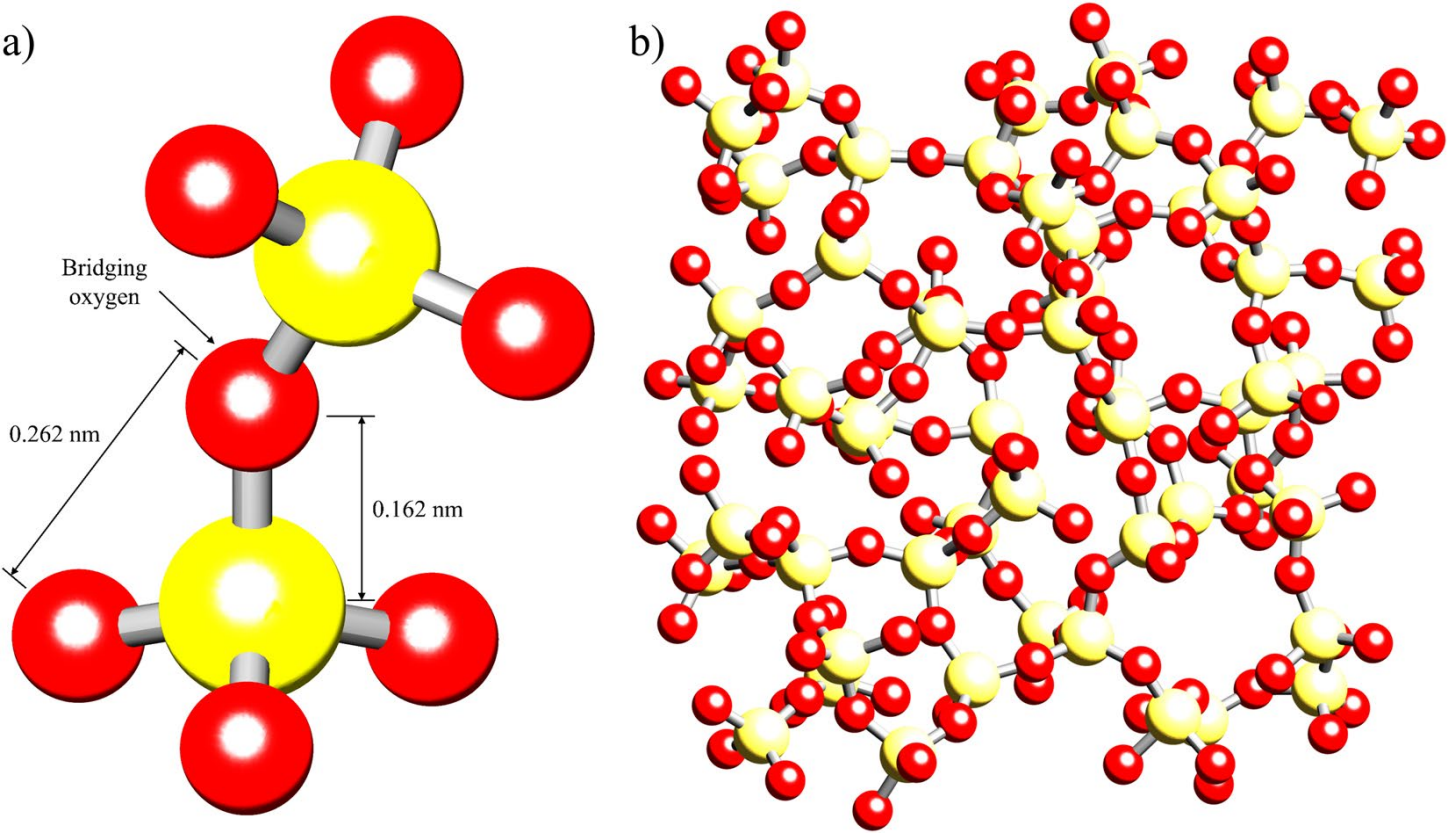
Atomic structure of amorphous silica. (**a**) Schematic representation of two tetrahedra showing nominal distances between neighbouring Si-O and O-O atoms, and a bridging oxygen between two neighbouring tetrahedra. (**b**) Subsection of an amorphous silica MD simulation showing a random arrangement of silica tetrahedra.

The structure of silica can be described as a system of SiO_4_ tetrahedra with a central silicon atom, surrounded by oxygen atoms at each vertex (Fig. 1a). In crystalline silica (such as quartz), the four oxygen atoms act as bridges to neighbouring tetrahedra, however in amorphous silica the arrangement of tetrahedra is random, so that not all oxygen atoms are bridging. The presence of non-bridging atoms allows the rotation of the individual tetrahedral with respect to its neighbours over a range of 120–180°, with minimal changes to the energy state within the system. This results in a break down in long range order, but also leads to preferential localised inhomogeneous deformation through bond rotation.

**Figure 2:**
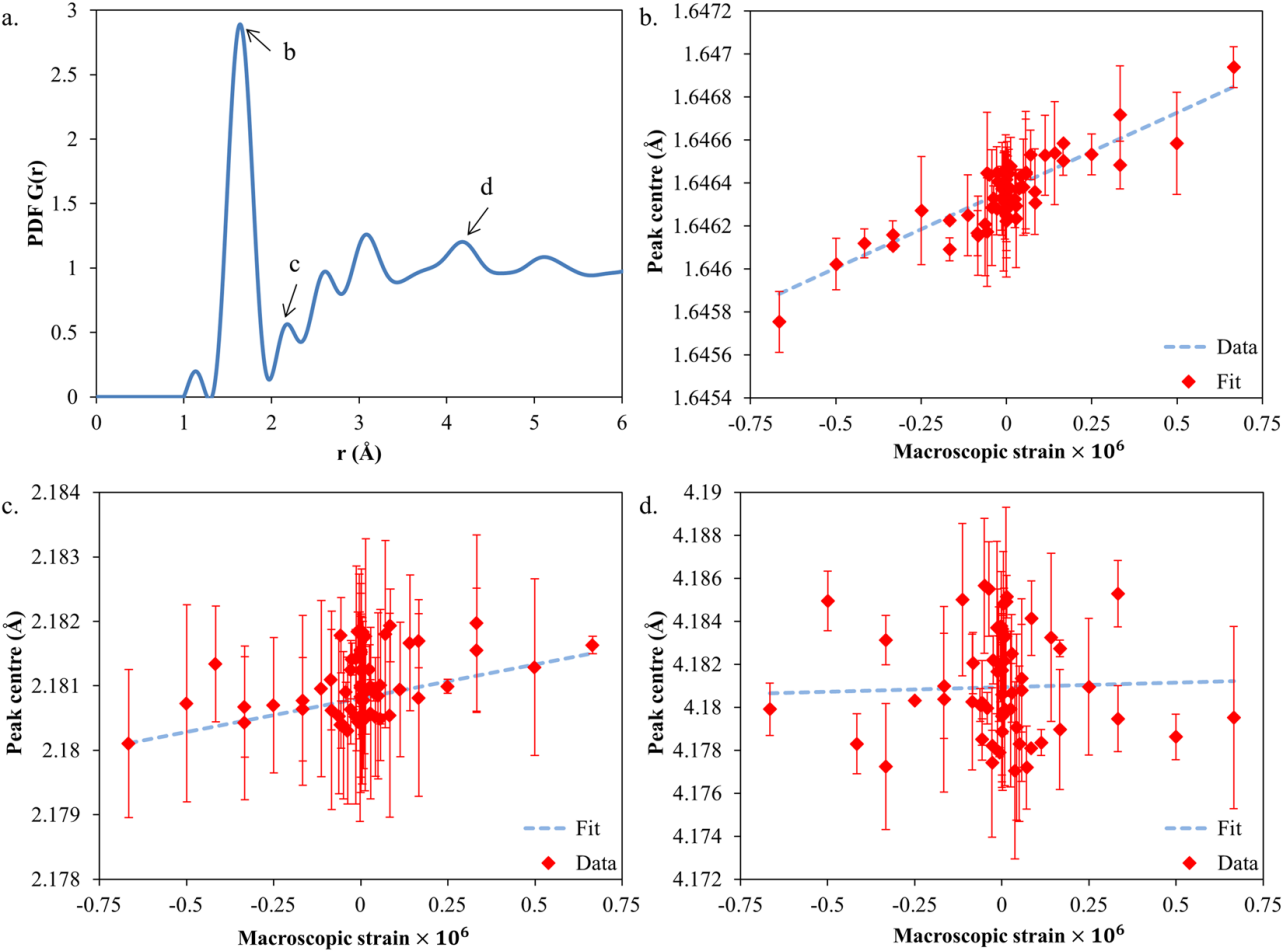
Variation of PDF peak centre with strain. (**a**) Typical PDF distribution of amorphous silica. Peak centre positions are shown as functions of macroscopic strain for peaks with (**b**) a strong correlation with applied strain (R = 0.88), (**c**) an intermediate level of correlation (R = 0.49) and (**d**) no correlation (R = 0.03).

The pair distribution function, *G*(*r*) (shown in Fig. 2(a) for silica) is a quantitative histogram of interatomic distances between a pair of atoms, with the peaks reflecting the coordination of the atoms^14^. The PDF provides structural information about the atomic environment, even for amorphous materials. Even in the absence of a complete structural model for a material, the deviations in the PDF peak positions and intensities provides a quantitative indication of the alteration in the local interatomic distances within the material.

**Figure 3:**
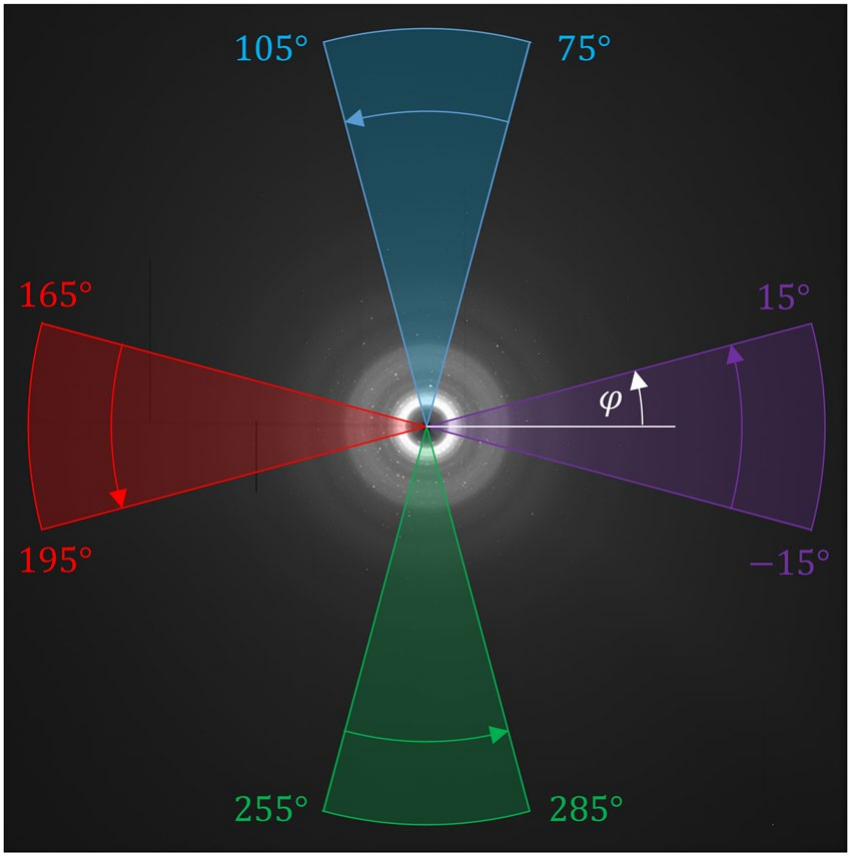
Amorphous silica diffraction pattern. The 2D diffraction pattern is azimuthally (φ) integrated over 30° to provide insight into the strain in the horizontal and vertical directions.

X-ray scattering data (Fig. 3) is collected for PDF analysis and interpreted in the form of the pair distribution function, *G*(*r*) (see Methods section). When macroscopic tensile or compressive strain is applied, peak centre positions change from ${r}_{0}^{k}$ to *r*^*k*^. The atomic strain ${\varepsilon }^{A}$ is calculated from the peak centre shift using the formula

1 $${\varepsilon }^{A}=\frac{{r}^{k}-{r}_{0}^{k}}{{r}_{0}^{k}},$$

where *r*^*k*^ and ${r}_{0}^{k}$ represented the strained and unstrained peak positions, respectively. The relationship between the applied macroscopic strain and the peak centre position is illustrated in Fig. 2(b–d).

There is a varying degree of consistency between the applied strain and peak shift (represented by *R*, the coefficient of determination), and the slopes are different. If an ideal correlation existed, one would expect to find *R*~1. In the coordinates used in Fig. 2, according to Equation (1) the slope of each graph should be close to ${r}_{0}^{k}$, the unstrained centre position of the *k*-th peak. In practice one finds strong deviations from the expected behaviour due to the combination of experimental noise and the complexity of the underlying deformation mechanisms.

**Figure 4:**
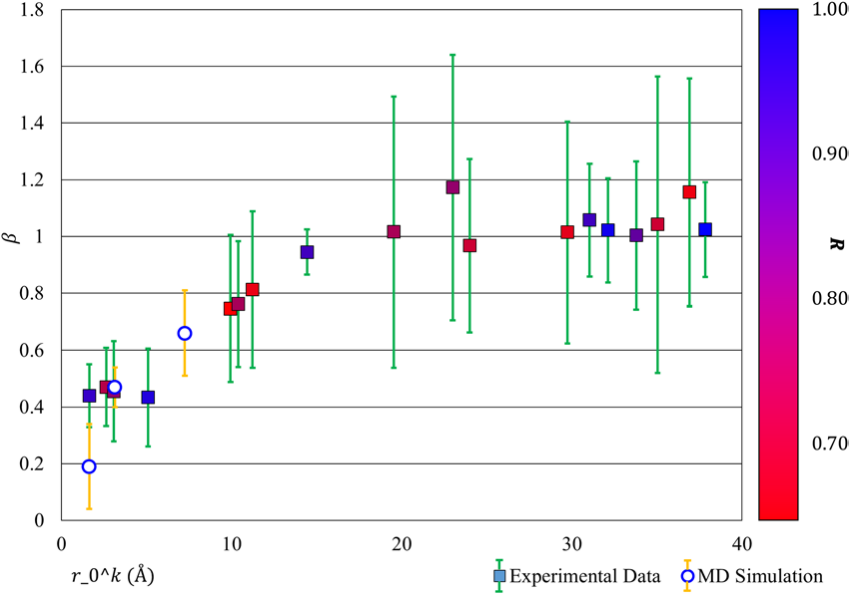
Relationship between atomic and macroscopic strains for different PDF peaks. The variation of the constant of proportionality between atomic and macroscopic strain (β) as a function of ${{r}}_{{\rm{0}}}^{{k}}$. The correlation coefficient between atomic and macroscopic strain (R) is indicated by the marker colour and the 95% confidence interval for each value is indicated by error bars.

In order to extract and interpret the prevailing trends in the observations, the plot shown in Fig. 4 was constructed in which the horizontal axis shows the peak centre position, whilst the vertical axis gives the ratio *β* between the observed atomic strain ${\varepsilon }^{A}$ and macroscopic strain ${\varepsilon }^{M}$ (see Equations (2) and (3) in the Methods section). Additionally, the colour of the marker for each data point represents the quality of correlation between the macroscopically applied strain and the relative peak shift, with blue markers denoting high quality of correlation (*R* ~ 1), and red markers indicating low correlation.

It is apparent that the strain ratio significantly lower than unity is observed for peaks with ${r}_{0}^{k}$ < 15 Å, whilst above this threshold even poorly correlated peaks show strain ratios close to or greater than 1. It is also apparent that in particular for peaks with *k* = 1 and *k* = 4, the quality of correlation is high, meaning that the mismatch between the local strain and macroscopic strain has statistical significance. If none of the nearest neighbour interatomic distances change by more than 50% of the imposed strain, how can the overall macroscopic deformation be accommodated?

In search for an explanation of this observation we performed molecular dynamics (MD) simulation of the deformation in amorphous silica, illustrated in Fig. 1, focusing our attention on the relative importance of bond stretching (changes in distance between nearest neighbours) *vs* bond angle variation that leads to the change in the distances between more remote atoms. Examination of the two nearest neighbour bond angles (O-Si-O and Si-O-Si) was performed in order to determine the prevalence of tetrahedral “stretching” through O-Si-O bond angle changes and tetrahedral “scissoring” through Si-O-Si bond angle changes. It became apparent that very significant changes in the Si-O-Si bond angle of up to tens of degrees are induced, whilst the changes in the O-Si-O bond angle and the bond lengths are noticeably smaller. To provide direct comparison with the experimental results, estimates of the *β* values for three selected peaks were extracted from MD simulations, and are shown in Fig. 4. It is clear that the trend observed for MD-based estimates shows a close match to the experimental data.

The observation of the importance of bond angle change in silica has been made previously by other authors in the context of electronic structure and chemical behaviour of silica^1^. Raman spectroscopy has also been performed to reveal the importance of bond angle changes in silica strain accommodation at high compressive stresses (multiple GPa)^15–17^. Despite this, little experimental evidence for the importance of this phenomenon in strain has previously been presented for tensile stresses or at the stress magnitudes examined in this study (<300 MPa)^18^. As well as corroborating these existing results, the insights gained from this diffuse diffraction study importantly reveal for the first time the 15 Å length scale over which this phenomenon influences the atomic strain response of silica. Further, our findings provide an explanation for the previously reported challenges in the use of PDF analysis for strain determination^6,12^, since whilst lower order peaks are easier to analyse, they show weak correlation with the macroscopic strain, with a knock-down ratio that needs to be calibrated either experimentally or through modelling for each individual system.

Interestingly, a similar local strain reduction factor of ~0.4 with respect to the macroscopic value has been reported before in attempts to interpret small angle X-ray scattering (SAXS) data^19^. In our previous study^20^ we proposed a resolution of this paradox through combined experimental and modelling deformation study of thermoplastic polyurethanes that revealed the non-affine nature of deformation due to structural inhomogeneity, the presence of “fuzzy interfaces” and their influence on the X-ray scattering peak formation^21^.

In conclusion, it has been shown for the first time that Atomic PDF analysis is a powerful tool which can be combined with MD simulations to provide insight into strain accommodation in amorphous materials. In this study, these two approaches have been used to show that strain accommodation within silica is dominated by tetrahedral scissoring rather than changes in bond length. The relationships between macro and atomic level strains demonstrate that atomic rearrangement, rather than bond stretching, is the dominating strain accommodation mechanism up to length scales of approximately 15 Å in this material.

## Methods

### Sample Preparation

The ceramic used for this study was VITABLOC^®^ Mark II which is a silica based dental porcelain manufactured by CEREC. A cuboidal four-point bending specimen was sectioned from the base material using a Buehler IsoMet^®^ Low Speed Diamond Saw. A progressively refined grinding and diamond polishing (1 μm grit) procedure was then applied to each surface to minimise the likelihood of localised stress concentration during loading. The final dimensions of the completed sample were 2 × 1.03 × 15.8 mm^3^. The through beam thickness was selected to be 2.00 mm in order to maximise the diffracted signal during synchrotron X-ray scattering.

### Synchrotron Experiment

X-ray scattering data was collected at beamline I15 at Diamond Light Source, UK, using an X-ray energy of 76 keV and a collimated beam with a circular cross-section and diameter of 70 μm. A Perkin Elmer flat panel 1621 EN area detector was placed 262 mm downstream from the sample in transmission geometry to facilitate data collection up to the maximum momentum transfer of $25.1{{\rm{\AA }}}^{-1}$. Calibration of the sample to detector distance was performed using lanthanum hexaboride (LaB_6_, NIST SRM 660b).

Optical microscopy was used to align the sample region of interest with the incident beam and Newport MFA-CC linear translation stages were used to move the sample. Data collection was implemented by collecting one minute of dark field data (which was used to correct for the detector background noise level), followed by one minute data collection. This sequence was repeated 12 times at each point on the sample in order to collect the statistics necessary to produce reliable pair distribution profiles. Background X-ray scattering data in the absence of the sample (flat field) was collected using the same data collection routine.

### *In situ* Four Point Bending

*In situ* four-point bending was performed on the cuboidal porcelain sample using a Deben Microtest 200 N loading rig. The loading direction was perpendicular direction to the incident beam and diffraction patterns were collected at the midpoint between the loading half cylinder pins. This arrangement provides a known moment and stress distribution as a function of the distance from the centre of the beam (*x*) and the applied load (*F*). At each of the three loading increments (*F* = 0 N,10 N and 20 N), ten scans were collected as a function of *x* position ranging from −0.45 mm to 0.45 mm in steps of 0.1 mm, which spanned the linear variation from tensile and compressively strained regions.

### Pair Distribution Function Analysis

The 2D X-ray scattering data was processed using the software package DAWN^22^ by initially averaging over the twelve sets of data collected at each point. In order to resolve the horizontal and vertical strain components, azimuthal (*φ*) integration of the resulting 2D data was performed over 30° sectors to give equivalent 1D distributions which were representative of each orientation as shown in Fig. 3. These two directions correspond to the strain induced by the applied force through bending as well as the perpendicular strains associated with the Poisson effect. A small number of crystalline Bragg peaks in the 2D data were observed and masked prior to processing using the tools available within DAWN. Rietveld refinement was used to estimate the volume fraction of this material, which was determined to be <1%. The segmented 1D scattering data was then corrected for background, Compton, and multiple scattering, and beam attenuation using the software Gudrun^23^ in order to produce the normalised total scattering function, *S*(*Q*). The total scattering function was then Fourier transformed to produce the PDF in the form of function *G*(*r*), as defined by Keen^24^.

### Macro vs Micro Strain Comparison

Least squares Gaussian peak fitting was applied to each of the 39 distinguishable peaks within the PDF radial distribution function (to a radial distance of $\sim 38\,{\rm{\AA }}$) in order to determine the peak centre and associated confidence interval at each position. Plots of the correlation between macroscopic strain and peak centre position were found to show varying levels of correlation coefficient (*R*) for different peaks as shown in Fig. 2c,d. Only those peaks with a strong level of correlation (*R* > 0.66) were selected. This gave 18 peaks, covering *r* values in the range 1.64–37.84 Å.

Least squares fitting was used to determine the relationship between the peak centre (*r*^*k*^) and macroscopic strain (${\varepsilon }^{M}$) for each of these peaks using:

2 $${r}^{k}=\alpha {\varepsilon }^{M}+{r}_{0}^{k}$$

where *α* is the coefficient relating the peak centre to the applied macroscopic strain. During this process the relative uncertainty of *r*^*k*^ was accommodated by applying a weighting factor equal to the inverse of the standard deviation obtained from peak fitting at each point. The standard deviations for *α* and ${r}_{0}^{k}$, *σ*_*α*_ and ${\sigma }_{{r}_{0}^{k}}$ respectively, were also determined.

Rearrangement of Equation 2 and substitution into Equation 1 reveals that the relationship between atomic (${\varepsilon }^{A}$) and macroscopic strain (${\varepsilon }^{M}$) can be written as:

3 $${\varepsilon }^{A}=\frac{\alpha }{{r}_{0}^{k}}{\varepsilon }^{M}=\beta {\varepsilon }^{M}$$

Here *β* is the constant of proportionality between the two measures of strain. Estimates of the standard deviation of *β*, *σ*_*β*_ were also obtained from the expression:

4 $${\sigma }_{\beta }=\frac{\alpha }{{r}_{0}^{k}}\sqrt{{(\frac{{\sigma }_{{r}_{0}^{k}}}{{r}_{0}^{k}})}^{2}+{(\frac{{\sigma }_{\alpha }}{\alpha })}^{2}}$$

in order to determine the confidence intervals of each value.

Further analysis was performed to assess the relationship between macroscopic strain and the other two peak descriptors used in the fitting process, Gaussian peak height and width. Low correlations were observed for these two measures for all of the peaks assessed.

### Molecular Dynamic Simulations

Molecular Dynamics (MD) simulations of amorphous silica were produced using the Atomistix ToolKit–Virtual NanoLab^25^. In order to create a representative starting model a large (1,728 silicon atoms and 3,456 oxygen atoms) unit cell of cristobalite was initially generated and melted to 5000 K using the ATK classical calculator and the silica potential developed by Pedone *et al*.^26^. It is important to highlight that although this potential is valid over the relatively low stresses (<300 MPa) applied in this study, simulation of the O-O soft shoulder would be required for higher pressure (multiple GPa) analysis^27^. The initial velocity of the atoms was based on a 5000 K Maxwell-Boltzmann distribution and 20,000 steps were used in the simulation. Next, a 20 step incremental cooling regime was simulated, during which the density of the amorphous material was monitored closely to ensure that it was representative of silica at room temperature (model = 2.27 *gcm*^−3^, actual = 2.2 *gcm*^−3^ ^28^). A tight bonding and annealing procedure was also implemented in order to minimise any residual stresses within the sample^29^ and to reduce the amount of defects within the amorphous material to realistic levels^30^. Examination of the resulting amorphous atomic structure revealed a random distribution of silicon surrounded by 4 oxygen atoms in the characteristic tetrahedral arrangement illustrated in Fig. 1b.

**Figure 5:**
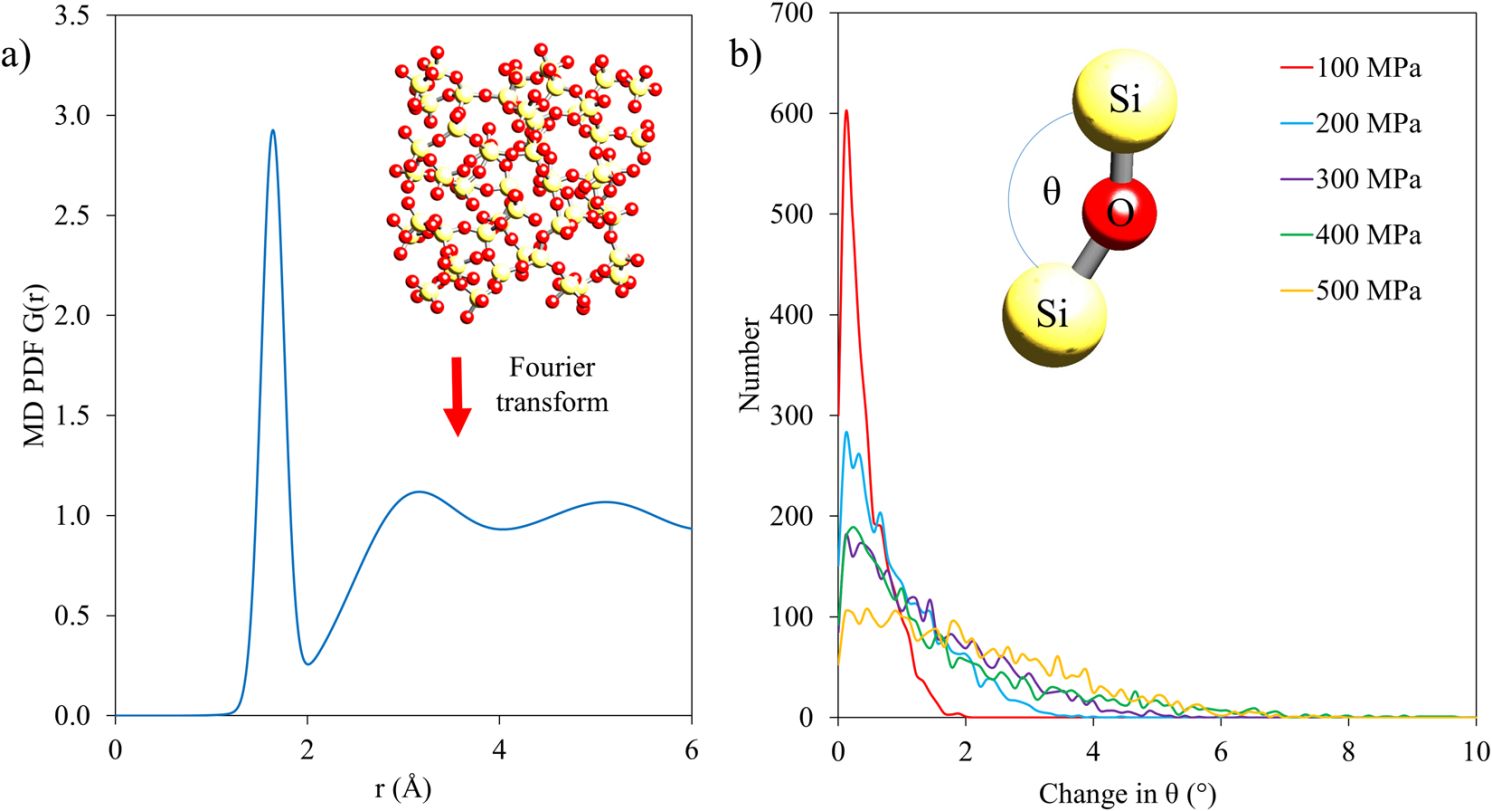
Results of amorphous silica MD simulations. (**a**) Example PDF generated from MD array. (**b**) Histograms of the reduction in θ (Si-O-Si bond angle) at the different loading pressures applied.

In order to simulate macroscopic straining of amorphous silica, an NPT Melchionna type simulation was used to apply external pressure to two opposing surfaces of the amorphous material. At the nanoscale lengths simulated in this analysis, this uniaxial simulation locally provides a good representation of the conditions induced by four point bending^31^. This assertion can be validated by quantifying the nominal stress difference expected between the top and bottom of the simulation volume based on the experimental geometry at the maximum load applied (20 N): a value of 3 kPa or 0.0009% of the stress expected at this location. A bulk modulus of 37 GPa^32^, and thermostat timescale of 20 fs was implemented in the simulation which was performed over 20,000 steps. To simulate the case of an unloaded sample, the first simulation was run at an applied pressure of 1 Pa. Pressures covering the range from 25 to 500 MPa were then incrementally applied in steps of 25 MPa to simulate the uniaxial loading. At each load the position of each atom was recorded, and a simulated PDF was generated as shown in Fig. 5a.

Critical examination of the atomic structure was performed to determine the origin of strain accommodation within the MD simulations. In particular, quantification of the changes to the Si-O bond length and the two nearest neighbour bond angles (Si-O-Si & O-Si-O) was performed. This analysis matched closely the results of the PDF peak shift analysis in that the average strain change in bond length was approximately 20% of that observed in the bulk. A histogram of the change in the Si-O-Si bond angle (θ) is shown in Fig. 5b, demonstrating that larger changes in the bond angle are present at higher loading pressures. Interestingly, the change of the O-Si-O bond angle was found to be on average an order of magnitude smaller than that of the Si-O-Si bond angle.

### Data Availability

Additional data can be accessed via ORA (http://ora.ouls.ox.ac.uk). Requests for any material samples or specimens described in this manuscript should be directed to the corresponding author.
